# A Cascade Bilayer Electron-Transporting Layer for Enhanced Performance and Stability of Self-Powered All-Inorganic Perovskite Photodetectors

**DOI:** 10.3390/molecules30102195

**Published:** 2025-05-17

**Authors:** Yu Hyun Kim, Jae Woong Jung

**Affiliations:** 1 Department of Advanced Materials Engineering for Information & Electronics, Kyung Hee University, 1732 Deogyeong-daero, Giheung-gu, Yongin-si 446-701, Gyeonggi-do, Republic of Korea; 2 Integrated Education Institute for Frontier Science & Technology (BK21 Four), Kyung Hee University, 1732 Deogyeong-daero, Giheung-gu, Yongin-si 446-701, Gyeonggi-do, Republic of Korea

**Keywords:** all-inorganic perovskite, photodetectors, ETLs, ZnO, SnO_2_, detectivity

## Abstract

This study aims to enhance optoelectronic properties of all-inorganic perovskite photodetectors (PDs) by incorporating a bilayer electron transport layer (ETL). The bilayer ETL composed of SnO_2_ and ZnO effectively optimizes energy level alignment at the interface, facilitating efficient electron extraction from the CsPbI_2_Br perovskite layer while suppressing shunt pathways. Additionally, it enhances interfacial properties by mitigating defects and minimizing dark current leakage, thereby improving overall device performance. As a result, the bilayer ETL-based PDs exhibit broadband photoresponsivity in 300 to 700 nm with a responsivity of 0.45 A W^−1^ and a specific detectivity of 9 × 10^13^ Jones, outperforming the single-ETL devices. Additionally, they demonstrate stable cyclic photoresponsivity with fast response times (14 μs for turn-on and 32 μs for turn-off). The bilayer ETL also improves long-term reliability and thermal stability, highlighting its potential for high performance, reliability, and practical applications of all-inorganic perovskite PDs.

## 1. Introduction

With the growing demand for optical signal detection and optical communication systems across a wide range of ultraviolet (250–400 nm), visible (450–800 nm), and infrared (900–1700 nm) wavelengths, photodetectors (PDs) have become essential elements in various optoelectronic applications [1,2,3]. Currently, most commercial PDs are fabricated using silicon (Si) or crystalline III–V semiconductors such as indium gallium arsenide (InGaAs) due to their broad spectral coverage, high mechanical stability, cost-effective scalability, and excellent spatial resolution [4]. However, their mechanical rigidity and vacuum-based complex fabrication processes hinder their integration into flexible, wearable, and deformable substrates and large-area devices [5,6,7,8,9]. Furthermore, state-of-the-art inorganic PDs often fail to meet the stringent performance requirements of modern applications, prompting extensive research into alternative photoactive semiconductors with enhanced photoresponse speed, sensitivity, and detectivity under low-light conditions [10].

Recently, organolead trihalide perovskites have garnered significant attention as promising semiconducting materials for a wide range of optoelectronic applications. Their exceptional properties, including high light absorption coefficients, long carrier diffusion lengths, and superior charge carrier mobilities, render them highly suitable for integration into solar cells and display technologies [11,12,13,14,15,16]. The perovskite semiconductors are also applied in PD applications as effective photoactive materials [17,18,19,20]. However, a major limitation of organic cation-based perovskites is their instability in humid environments and high thermal/electrical stress, which poses a significant challenge for their practical implementation in real-world uses [21]. To address the stability issue, all-inorganic lead halide perovskites, particularly cesium-based compositions (CsPbX_3_, where X = I, Br, Cl), have been extensively investigated as potential alternatives due to their superior environmental resilience [22]. These perovskites exhibit remarkable optoelectronic properties, including a high light absorption coefficient (>10^5^ cm^−1^) and impressive charge carrier mobility (~1000 cm^2^ V^−1^ s^−1^), comparable to silicon. As a result, they have garnered significant attention for application in photodetectors (PDs) [23]. For instance, CsPbBr_3_ microwire-based PDs have been demonstrated to achieve an obvious responsivity improvement of 145% [24]. Shoaib et al. synthesized ultralong CsPbBr_3_ nanowire-based PDs with a responsivity of 4.4 × 10^3^ A W^−1^ and a response speed of 0.252 ms, highlighting their potential for advanced optoelectronic applications [25].

Despite these promising advancements, the operational stability and reliability of all-inorganic perovskite-based PDs remain hindered, particularly when organic interface materials are incorporated into device architectures. Typically, organic *p*-type hole-transporting materials such as poly(3,4-ethylenedioxythiophene):poly(styrenesulfonate) (PEDOT:PSS) and poly[bis(4-phenyl)(2,4,6-trimethylphenyl)amine] (PTAA) are utilized in perovskite PDs with a *p-i-n* structure [26]. Conversely, *n-i-p* architectures often integrate inorganic electron-transporting layers (ETLs), including zinc oxide (ZnO) or tin(IV) oxide (SnO_2_), which provide high mechanical stability and long-term reliability of the interface in devices [27]. These inorganic *n*-type oxide semiconductors support a stable foundation for perovskite layers, thereby enhancing device performance and stability. However, a key challenge in *n-i-p* perovskite PDs with *n*-type oxide semiconductors is balancing strong photoresponse with low dark-state current, because the inherently high conductivity of the oxide semiconductors can lead to excessive dark current, ultimately degrading the on/off ratio of PDs [28]. To compensate for those restrictions, a bilayer structure ETL has been designed to improve the electrical properties of single-layer *n*-type oxide semiconductor-based devices. Sun et al. have incorporated ZnO into the TiO_2_ ETL to regulate the grain size and improve the surface uniformity of the perovskite absorber layer in photovoltaic devices [29]. A bilayer ETL structure comprising SnO_2_ and ZnO has also been developed for high-performance perovskite solar cells, enabling a tunable electronic structure for efficient electron extraction [30]. However, bilayer ETLs composed of different *n*-type metal oxides have not been extensively investigated in perovskite photodetectors, despite their potential to provide favorable energy level alignment with perovskite absorbers for self-powered operation.

In this study, we designed a bilayer inorganic ETL comprising SnO_2_ and ZnO for integration into high-performance all-inorganic perovskite PDs, aiming to enhance stability and operational reliability. This bilayer ETL design leverages the high electrical conductivity of ZnO to promote efficient electron transport, thereby improving charge extraction and transfer efficiency. As a result, the bilayer ETL-based PDs exhibited notable performance enhancements, achieving a responsivity that optimized charge extraction while suppressing dark-state current. The resulting PDs achieve a responsivity of 0.45 A W^−1^ at 630 nm, a specific detectivity of 9 × 10^13^ Jones, and a fast response time of less than 50 μs. These improvements surpass those observed in devices utilizing a single ETL layer (ZnO or SnO_2_) in all-inorganic perovskite PDs. Furthermore, the optimized bilayer ETL-based PDs maintained excellent stability for a 14-day period, demonstrating their potential as a viable pathway toward highly stable and reliable PDs with superior optoelectronic performance.

## 2. Results and Discussion

**Figure 1:**
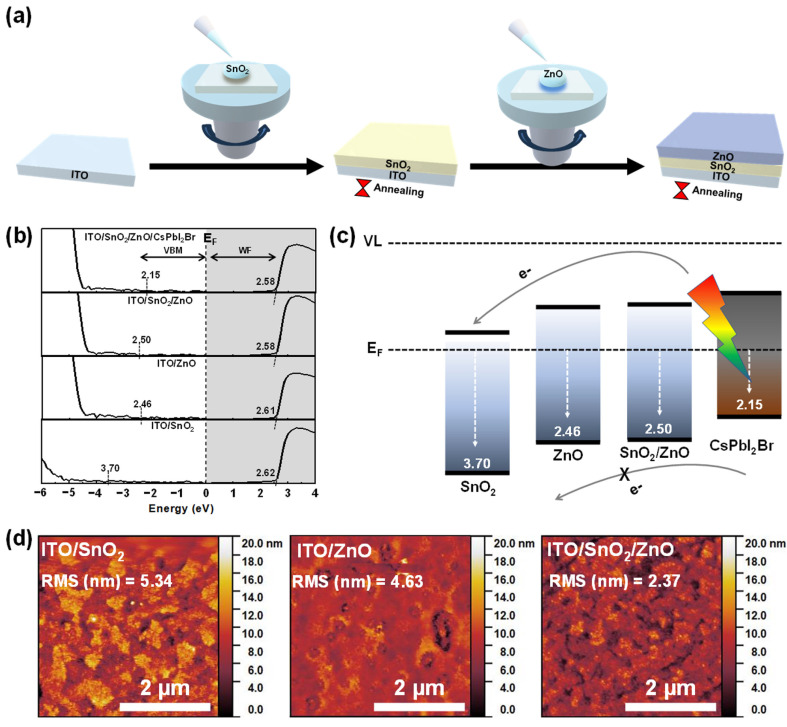
Fabrication process of ETLs (**a**), UPS binding energy spectra (**b**), energy diagram (**c**), and AFM topographic images (**d**) of single-layer and bilayer ETLs.

To construct a bilayer ETL, SnO_2_ and ZnO layers were sequentially deposited onto an ITO substrate. Figure 1a illustrates the fabrication process of the bilayer ETL, where SnO_2_ and ZnO thin films were formed via spin-coating technique. The ZnO and SnO_2_ precursor solutions were applied successively, followed by a thermal annealing process. The detailed procedure for preparing the bilayer ETL is provided in the Experimental section. To analyze the energy level alignment of each ETL layer, ultraviolet photoelectron spectroscopy (UPS) measurements were conducted. Figure 1b presents the secondary electron cut-off and valence band (VB) region, extracted from the UPS binding energy spectra of the samples. The VB maximum (VBM) value of each ETL was determined from the onset of the binding energy spectrum where the intensity rises above the noise baseline, as indicated in each spectrum in Figure 1b. It was observed that the work function (WF) of each ETL deposited on ITO was approximately 2.6 eV. However, the VBM values exhibited significant modulation: SnO_2_ had a VBM of 3.70 eV, whereas ZnO showed a VBM of 2.46 eV, indicating a substantial vacuum level (VL) shift. This pronounced VL modulation can be attributed to the differing electron transfer capabilities of the materials, with SnO_2_ demonstrating a stronger electron transfer to ITO compared to the ZnO layer. When constructing the bilayer ETL with ZnO/SnO_2_, the VBM of the ITO/ETL structure was determined to be 2.50 eV, closely resembling that of a single ETL of ZnO film. This configuration enables stepwise energy level alignment at the interface between the ITO and CsPbI_2_Br perovskite layers; therefore, the inverted bilayer ETL structure (SnO_2_/ZnO) is not favorable for efficient electron transport and extraction from the perovskite photo-harvesting layer. The resulting stepwise band alignment effectively enhances the energy cascade matching with CsPbI_2_Br films, thereby reducing electron recombination, thermionic losses, and current leakage at the perovskite/ETL interface. Furthermore, considering the wide optical bandgap of oxide semiconductors (SnO_2_~3.7 eV and ZnO~3.3 eV), the conduction band (CB) of the bilayer ETL provides a favorable electron extraction channel from the CsPbI_2_Br perovskite to the ITO cathode [27]. This configuration minimizes energetic disorder across the interfaces between the ITO and perovskite layers, as depicted in Figure 1c.

The surface topographical characteristics of the bilayer ETL in comparison to the single ETL were investigated using atomic force microscopy (AFM). As shown in Figure 1d, the SnO_2_ layer deposited on ITO exhibited a rough film with large grain structures, reflecting the surface morphology of the underlying ITO substrate. Similarly, the ITO/ZnO sample also displayed a rough surface with prominent grain formations due to the influence of the bottom ITO layer. In contrast, the bilayer ETL film demonstrated a more uniform and smoother surface, indicating that the bilayer structure effectively covers the roughness of the underlying substrate. The highly uniform and compact morphology is beneficial for reducing leakage current at the device interfaces, which in turn lowers the dark current, a critical factor in enhancing the responsivity of photodetectors. The root-mean-square (RMS) roughness values of the surfaces were measured to be 5.34 nm and 4.63 nm for the ITO/SnO_2_ and ITO/ZnO samples, respectively. Notably, the ITO/SnO_2_/ZnO bilayer film exhibited a significantly reduced RMS roughness of 2.37 nm. This substantial decrease in surface roughness suggests that the bilayer ETL promotes the formation of a highly compact and defect-minimized film, which can enhance electron transport efficiency and suppress non-radiative recombination. As a result, improved electrical properties are anticipated for the bilayer ETL-based devices. Since the largely aggregated nanoparticles could act as a barrier for homogeneous growth of the perovskite absorber, well-passivated surfaces of the bilayer ETLs could facilitate uniform nucleation and growth of the perovskite absorber. In addition, minimized shunt pathways and corresponding suppressed trap-assisted recombination at the interfaces could be expected in the bilayer ETL-based device [31].

**Figure 2:**
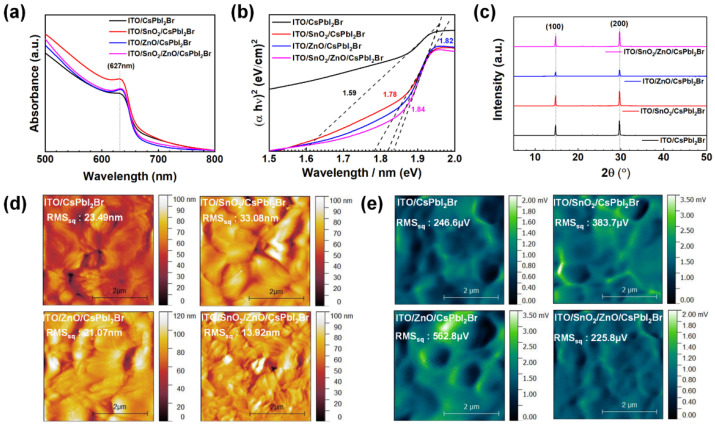
Absorption spectra (**a**), Tauc plots (**b**), XRD patterns (**c**), AFM topographic images (**d**), and KPFM images (**e**) of CsPbI_2_Br films based on varied ETL-based substrates.

Figure 2a displays the absorption spectra of perovskite films grown on different ETL substrates. All films exhibited a characteristic absorption peak at 627 nm, typical of CsPbI_2_Br, with variations in the absorption tail near 700 nm. The Tauc plots of the four samples reveal that the absorption edge shape is strongly associated with electronic transitions in the band-edge region. As shown in Figure 2b, a reduced optical bandgap observed in the Tauc plots suggests the presence of excitonic tail states, likely due to a significant density of defects in the CsPbI_2_Br films. The bandgap of the CsPbI_2_Br film on bare ITO was 1.59 eV, whereas films grown on ETLs exhibited slightly higher bandgaps of approximately 1.78 eV and 1.82 eV for ITO/SnO_2_ and ITO/ZnO, respectively. Notably, the bilayer ETL resulted in the widest bandgap, indicating a more stable electronic structure with lower defect density compared to films grown on single ETL-based substrates.

The crystalline structure of the perovskite films was further analyzed using X-ray diffraction (XRD). Intense diffraction peaks were observed at 2θ ~12.6° and 2θ ~29.7°, corresponding to the (1 0 0) and (2 0 0) facets of CsPbI_2_Br lattices. The average crystallite size was determined using the following Debye–Scherrer equation:

(1) Dhkl=0.89λβ cosθ

where *D*_(*hkl*)_ is the average crystal size for the (*h k l*) facet, *λ* is the wavelength of X-ray (0.154 nm), *θ* is the Bragg diffraction angle, and *β* is the full width at half maximum (FWHM) of the peak. While the peak positions of the (1 0 0) and (2 0 0) reflections remained nearly identical across all samples, the FWHM of the (2 0 0) peak was narrowest in the CsPbI_2_Br films grown on bilayer ETL substrates, suggesting improved crystallinity. Additionally, the XRD peaks were most intense in films grown on bilayer ETL-based substrates, further confirming the highest degree of crystallinity. These findings align well with the Tauc plot analysis, as shown in Figure 2c.

To assess the impact of different ETL substrates on the topographical characteristics of perovskite films, AFM and Kelvin probe force microscopy (KPFM) were performed on CsPbI_2_Br films, as shown in Figure 2d,e. Notably, films deposited on rough substrates, including bare ITO, ITO/SnO_2_, and ITO/ZnO, exhibited significant surface roughness. In contrast, perovskite films grown on the bilayer ETL demonstrated a relatively smoother morphology, attributed to the uniform surface properties of the bilayer ETL film. Additionally, KPFM measurements revealed variations in the contact potential difference across the perovskite films. For samples prepared on ITO, ITO/SnO_2_, and ITO/ZnO, substantial potential barriers were observed at the grain boundaries, indicating non-uniform charge distribution. However, the bilayer ETL substrate (ITO/SnO_2_/ZnO) facilitated a more homogeneous and smooth potential distribution across the perovskite film. These results further confirm that rougher bottom layers do not provide an ideal platform for the growth of highly crystalline and uniform perovskite films. Conversely, the bilayer ETL, with its compact and pinhole-free surface, enables the formation of high-quality CsPbI_2_Br films, which are beneficial for efficient charge transport and reduced charge carrier recombination in perovskite PD applications.

**Figure 3:**
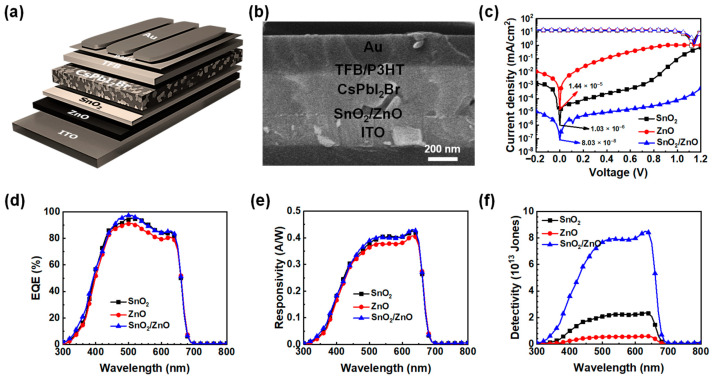
Schematic images (**a**) and cross-sectional SEM image (**b**) of the device with a bilayer ETL. *J–V* curves (**c**), EQE spectra (**d**), responsivity spectra (**e**), and specific detectivity spectra (**f**) of the devices prepared with varied ETL substrates.

To characterize device performance of the perovskite PDs, *n-i-p* planar heterojunction devices (ITO/ETL/CsPbI_2_Br/TFB/P3HT/Au) were fabricated, as illustrated in Figure 3a. Details of the device fabrication condition are described in the Experimental section. A cross-sectional SEM image of a representative device incorporating a bilayer ETL (SnO_2_/ZnO) is shown in Figure 3b, demonstrating a well-defined layered structure. The thickness of each ETL was measured to be approximately 20–30 nm, and the total thickness of the bilayer ETL in the optimized condition is estimated to be around 50 nm, using a surface profilometer. A bilayer ETL thicker than 50 nm led to reduced device performance, characterized by an increase in dark current and a decrease in EQE, compared to the optimized condition (Figure S1). The current density–voltage (*J–V*) characteristics of the ETLs with optimized conditions, recorded in both dark conditions and under illumination (100 mW cm^−2^, AM 1.5G) across a bias range of −0.2 V to 1.2 V, are presented in Figure 3c. The photocurrent density generated from the CsPbI_2_Br-based PDs was comparable regardless of the ETL structure, and the open-circuit voltage (V_OC_) was approximately 1.15 V, attributed to the large band gap of the CsPbI_2_Br perovskite absorber layer. For single ETL devices, the dark current density at zero bias ranged between 10^−5^ and 10^−6^ mA cm^−2^, which further dropped to 10^−8^ mA cm^−2^ with the incorporation of the bilayer ETL. This reduction is primarily attributed to minimized interfacial leakage current and a decrease in perovskite film defects, both of which stem from the favorable stepwise energy level alignment and the compact surface morphology, as discussed above. The suppressed leakage current in bilayer ETL-based devices was further verified through space charge limited current (SCLC) measurements in electron-only devices, as outlined in the Supplementary Materials (Figure S2). The trap-filled limit voltage (*V*_TFL_) was measured at 0.70 V and 0.39 V for SnO_2_- or ZnO-based devices, respectively, whereas the bilayer ETL device exhibited a substantially lower *V*_TFL_ of 0.30 V. Correspondingly, the trap density (*N*_T_) of the single ETL-based device was calculated to be 5.37 × 10^15^ cm^−3^ and 2.99 × 10^15^ cm^−3^ for SnO_2_ and ZnO, respectively, and the bilayer ETL mitigates the trap density to 2.30 × 10^15^ cm^−3^, confirming that the bilayer ETL effectively suppresses trap-assisted recombination and stabilizes the current flow in CsPbI_2_Br-based PDs (Table S1).

To assess the influence of different ETLs on the photodetection performance of perovskite PDs, the *EQE* spectra were analyzed. As illustrated in Figure 3d, all three devices demonstrated strong photoresponse within the 300–700 nm wavelength range. Notably, the *EQE* exceeded 90% in the 450–650 nm region for three devices, suggesting that highly crystalline CsPbI_2_Br perovskite films were successfully deposited on each ETL. However, the bilayer ETL exhibited the highest photoresponse characteristics among the three devices. Based on the *EQE* spectra, the spectral responsivity, a key parameter reflecting the efficiency of photodetection, was calculated using the following Equation (2):

(2) Rλ (AW−1)=JphI=EQE⋅λ1240

where *J_ph_*, *I*, and *EQE* denote the photocurrent density, the light intensity, and the *EQE* value at a given wavelength (*λ*), respectively [32]. As shown in Figure 3e, all devices operated at zero bias and exhibited a broad UV–vis photoresponse (300–700 nm). The bilayer ETL-based PDs achieved a peak spectral responsivity of 0.45 A W^−1^ at 630 nm, surpassing the 0.41 A W^−1^ recorded for single ETL-based devices. Additionally, the specific detectivity (D^*^), a critical metric for evaluating photodetector sensitivity, particularly under low-light conditions, was determined under the assumption that shot noise dominates. The detectivity was calculated using the following Equation (3):

(3) D*(cm Hz12W−1 or Jones)=R⋅A122⋅e⋅Jdark12

where *R*, *A*, *e*, and *J_dark_* represent the spectral responsivity, the effective area of the device, the elementary charge, and the dark current density [33]. The SnO_2_-based PDs achieved a detectivity of 2.5 × 10^13^ Jones, while the ZnO-based devices reached 6× 10^12^ Jones. Notably, the bilayer ETL-based PDs exhibited an enhanced detectivity of 9 × 10^13^ Jones, significantly outperforming their single ETL counterparts at zero bias. This improvement is attributed to the substantially lower dark current in the bilayer ETL-based device.

**Figure 4:**
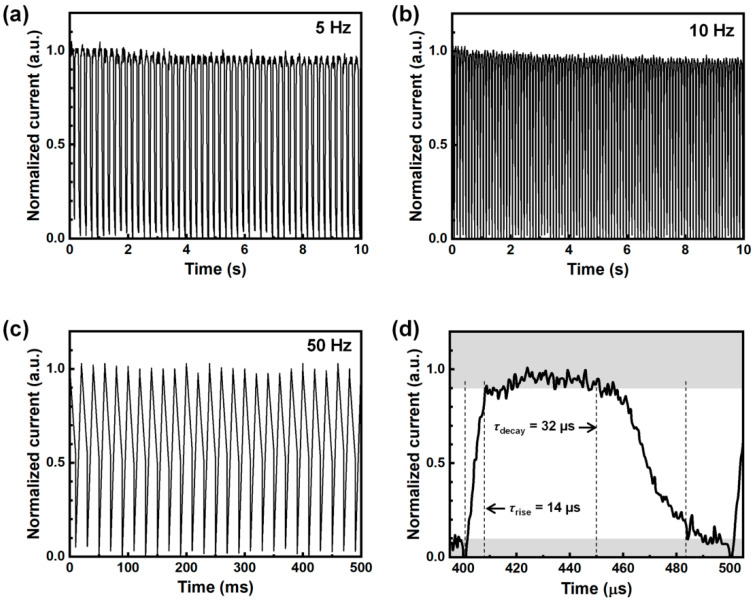
Temporal current response of PD devices with varied light frequencies of 5 Hz (**a**), 10 Hz (**b**), and 50 Hz (**c**), and magnified current response at “on” and “off” states of the device (**d**).

Given the excellent photoresponse properties of the perovskite PDs with an ETL of SnO_2_/ZnO, we further explored the temporal response behavior of the optimized devices employing a bilayer ETL. The time-resolved photoresponse of the passivated PD was measured under white light illumination at an intensity of 3 mW cm^−2^, with no applied electrical bias and varying light on-off frequencies, as illustrated in Figure 4a–c. As the modulation frequency increased from 5 Hz to 10 Hz and 50 Hz, the device maintained a consistent photoresponse, which presents stable and reproducible photoswitching behavior with rapid on/off response. To assess the response speed, the time-resolved photocurrent at 50 Hz was analyzed by magnifying the “*on*” and “*off*” states, as shown in Figure 4d. The photocurrent displayed a sharp transition between the two states, indicating a swift reaction to light illumination. The response and recovery times, which define the time required for the dark current to reach 90% of the maximum photocurrent and to drop to 10%, were estimated to be 14 μs and 32 μs, respectively. These values demonstrate the exceptional capability of perovskite PDs to detect high-speed optical signals, making them ideal for high-performance optoelectronic applications. The rapid photoresponse performance of the bilayer ETL-based perovskite PDs can be attributed to the facilitated electron transport properties with minimized charge recombination at the ETL interfaces, as well as high-quality CsPbI_2_Br perovskite films. This improvement facilitates efficient charge transport while minimizing recombination losses, thereby enhancing overall device performance.

**Figure 5:**
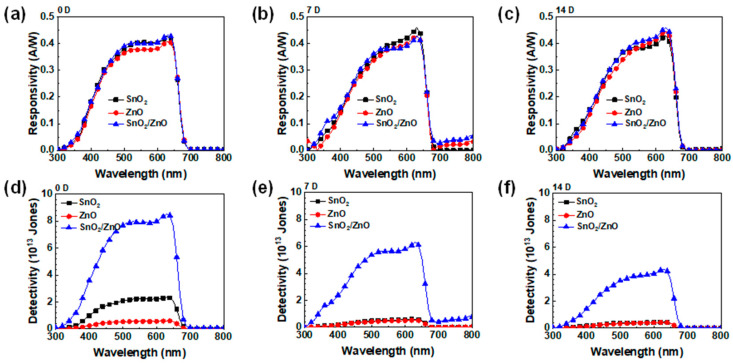
Responsivity (**a**–**c**) and specific detectivity (**d**–**f**) of PD devices with varied ETLs for fresh devices (**a**,**d**) and aged devices after 7 days (**b**,**e**) and 14 days (**c**,**f**) stored in ambient atmosphere.

Finally, we evaluated the stability of the bilayer ETL-based perovskite PDs, as long-term durability is crucial for practical applications. Perovskite semiconductors often face stability challenges when used as photoharvesting layers in PDs. To assess stability, the photoresponse characteristics of freshly prepared devices were measured after storage for 7 and 14 days. As shown in Figure 5a, the responsivity of the perovskite PDs remained stable over 14 days across all ETLs; however, the specific detectivity gradually decreased during the same period. Notably, PDs employing a single ETL exhibited a continuous decline in detectivity, with almost no detectable performance after 7 days (Figure S3 for dark J-V curves and EQE spectra for 2 weeks). In contrast, the bilayer ETL-based device retained over 80% of its original detectivity after 1 week and more than 50% after 2 weeks, indicating significantly improved long-term stability. Furthermore, when subjected to continuous heating at 80 °C, the devices preserved under ambient conditions for 2 h maintained more than 50% of their initial photoresponse (Figures S4 and S5). This suggests that CsPbI_2_Br perovskite PDs with a bilayer ETL are robust against thermal stress. Enhanced stability is primarily attributed to the bilayer ETL structure, which effectively protects the perovskite layer from moisture and oxygen during continuous heating or prolonged storage under ambient conditions. These results underscore the promising potential of bilayer ETL for improving the performance and durability of self-powered perovskite-based photodetectors, particularly for energy-efficient sensing applications such as environmental monitoring, biomedical diagnostics, and wearable technologies.

## 3. Experimental Procedure

### 3.1. Materials

The chemicals and solvents were purchased from commercial sources and used as received without further purification. Cesium iodide (CsI, 99.99%), lead(II) iodide (PbI_2_, 99.99%), and lead(II) bromide (PbBr_2_, 99.99%) were purchased from TCI (Tokyo, Japan). ZnO nanoparticles (nanopowder), ammonium hydroxide (98%), chlorobenzene (CB, anhydrous, 99.8%), chloroform (CF, anhydrous, 99.8%), N,N-dimethylformamide (DMF, anhydrous, 99.8%), and dimethyl sulfoxide (DMSO, anhydrous, 99.8%) were purchased from Sigma-Aldrich (St. Louis, MO, USA). Tin(IV) oxide (SnO_2_ colloidal dispersion in H_2_O, 15%) was purchased from Alfa aesar (Haverhill, MA, USA). Poly(3-hexylthiophene) (P3HT, MW~42 kDa) and poly[(9,9-dioctylfluorenyl-2,7-diyl)-*co*-(4,4′-(N-(4-sec-butylphenyl)diphenylamine)] (TFBI, MW~10 kDa) were purchased from Lumtec (New Taipei City, Taiwan).

### 3.2. Device Fabrication

ITO-coated glass substrates were cleaned with successive ultrasonication in deionized water, acetone, and isopropanol for 30 min, and then those were dried by N_2_ gas blowing. After the ITO substrates were treated with UV–Ozone for 15 min, the PEDOT:PSS layer was deposited by spin-coating the aqueous solution at 4000 rpm for 60 s. The films were annealed at 150 °C for 30 min to evaporate the water. After the substrates were cooled down to room temperature, they were transferred to an N_2_-filled glove box. The SnO_2_ layer was deposited by spin coating of the SnO_2_ dispersion solution by spin-coating at 3000 rpm for 30 s, followed by thermal annealing at 100 °C for 30 min. For ZnO deposition, the precursor solution was prepared by dissolving 80 mg of ZnO nanopowder in 12 mL of aqueous ammonium hydroxide solution (1.2 wt%). The ZnO precursor solution was deposited by spin-coating at 3000 rpm for 30 s, followed by thermal annealing at 100 °C for 30 min. The CsPbI_2_Br precursor solution, which was prepared by mixing CsI, PbBr_2_, and PbI_2_ (2:1:1 in molar ratio) in DMF and DMSO (2:1 in volume ratio), was spin-coated onto ETL substrates at 2500 rpm for 45 s. The substrates were then sequentially annealed at 50 °C for 2 min, 100 °C for 2 min, and 300 °C for 10 min, as reported elsewhere [34]. The TFB layer was formed by spin-coating the TFB solution (2 wt% in CB) at 5000 rpm for 30 s and then annealing at 100 °C for 10 min. The P3HT solution (1 wt% in CF) was dynamically spin-coated on TFB film at 3000 rpm for 30 s, followed by annealing at 100 °C for 10 min. Finally, Au (80 nm) was deposited by thermal evaporator under the vacuum system (~10^−6^ Torr) to define the active area of the device (10 mm^2^).

### 3.3. Characterizations

Photoelectron spectroscopies were carried out using X-ray photoelectron spectroscopy (K Alpha, Thermo Science, Waltham, MA, USA) and ultraviolet photoelectron spectroscopy (AXIS Ultra DLD, Kratos, Inc., San Diego, CA, USA) equipped with a He I source (hv = 21.2 eV) at the Core Facility Center for Analysis of Optoelectronic Materials and Devices of Korea Basic Science Institute (KBSI). The film surface morphologies were measured by field emission scanning electron microscopy (FE-SEM) (MERLIN, Carl Zeiss, Oberkochen, Germany) and atomic force microscopy (AFM) (CoreAFM, Nanosurf, Liestal, Switzerland). The crystallinity of the perovskite layer was measured by using X-ray diffraction (XRD) with a MiniFlex 300 (Rigaku, Tokyo, Japan). The optical spectra of the films were measured by UV–vis spectrophotometer (Cary100, Agilent, Santa Clara, CA, USA) and fluorescence spectrometer (FS5, Edinburgh Instruments, Livingston, UK). The picosecond laser with a wavelength of 405 nm (EPL-405, Edinburgh Instrument) was used in transient photoluminescence measurement. The electrical properties of the devices were obtained by a sourcemeter (4200-SCS, Keithley, Cleveland, OH, USA). The external quantum efficiency (EQE) of the device was measured with a chopped monochromatic light that is activated by a lock-in amplifier system in the short-circuit condition.

## 4. Conclusions

In conclusion, we examined the impact of a bilayer ETL structure on CsPbI_2_Br perovskite layers in self-powered perovskite PDs. To enhance the electronic properties and durability of the *n*-type ETLs, a bilayer configuration consisting of SnO_2_ and ZnO was employed, which were deposited using the spin coating method. This bilayer ETL successfully smooths the surface, preventing direct contact and the formation of shunt pathways between the cathode and perovskite interfaces. Furthermore, the bilayer ETL features energy levels that facilitate efficient electron transport while simultaneously blocking hole carriers at the interface between the ETLs and the CsPbI_2_Br perovskite layer. This configuration supports rapid and efficient electron transfer from the perovskite absorber to the cathode of the device. As a result of these optimizations, devices with fullerene passivation exhibited a significant reduction in dark current density and improved EQE across the spectral range of 300 to 700 nm. These enhancements stemmed from the ETL structure’s direct influence on the excellent photodetection performance of perovskite PDs, with the devices demonstrating high responsivity values exceeding 0.45 A W^−1^ and a detectivity as high as 9 × 10^13^ Jones during self-powered operation (zero bias voltage). The bilayer ETLs enabled rapid photoresponse behavior in the perovskite PDs, with fast turn-on/turn-off behavior under zero bias conditions. Moreover, the bilayer ETL demonstrated exceptional long-term reliability and high thermal stability of its photodetecting characteristics, which presents the potential of bilayer ETL for practical perovskite PDs with high device performance and reliability.

## Data Availability

The original contributions presented in this study are included in the article/Supplementary Material. Further inquiries can be directed to the corresponding author.

## Supplementary Materials

The following supporting information can be downloaded at: https://www.mdpi.com/article/10.3390/molecules30102195/s1, Table S1: Summary of SCLC measurements.; Figure S1: Dark *J*-*V* curves and EQE spectra of the bilayer ETL-based PD devices with varied ETL thickness.; Figure S2: SCLC electron-only devices of CsPbI_2_Br layer with varied ETLs.; Figure S3: Dark *J*-*V* curves and EQE spectra of PD devices for fresh and aged devices; Figure S4: Dark *J*-*V* curves and EQE spectra of PD devices with varied ETLs for fresh and thermally annealed devise; Figure S5: Responsivity and specific detectivity of PD devices with varied ETLs for fresh and thermally annealed devices.
