# Supplementary material for: A Cascade Bilayer Electron-Transporting Layer for Enhanced Performance and Stability of Self-Powered All-Inorganic Perovskite Photodetectors
Source: Molecules. 2025 May 17;30(10):2195. doi: 10.3390/molecules30102195 (PMC12113987; doi:10.3390/molecules30102195)
Supplement: Supplementary file 1 [file molecules-30-02195-s001.zip › molecules-3598202-supplementary.pdf]

Supplementary Data

A Cascade Bilayer Electron-Transporting Layer for Enhanced Performance and Stability of Self-Powered All-Inorganic Perovskite Photodetectors

Yu Hyun Kim [1,2] and Jae Woong Jung [1,2,*]

[1] Department of Advanced Materials Engineering for Information & Electronics, Kyung Hee University, 1732 Deogyeong-daero, Giheung-gu, Yongin-si 446-701, Gyeonggi-do, Republic of Korea

[2] Integrated Education Institute for Frontier Science & Technology (BK21 Four), Kyung Hee University, 1732 Deogyeong-daero, Giheung-gu, Yongin-si 446-701, Gyeonggi-do, Republic of Korea

* Correspondence: wodndwjd@khu.ac.kr

Table S1. Summary of V_{TFL} and N_t obtained from SCLC measurements.

ETL	V_{TFL} (V)	N_t (10^{15} cm^{-3})
SnO_2	0.70	5.37
ZnO	0.39	2.99
SnO_2/ZnO	0.30	2.30

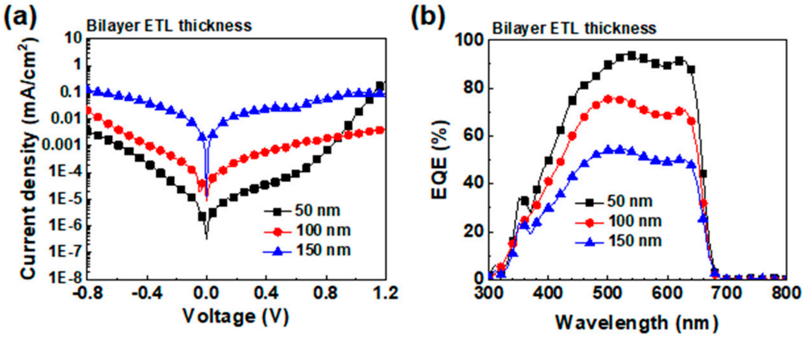

Figure S1. Dark *J-V* curves (a) and EQE spectra (b) of the bilayer ETL-based PD devices with varied ETL thickness.

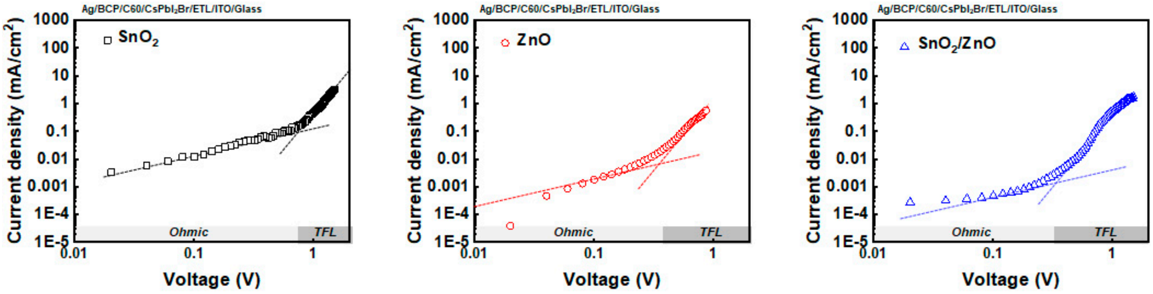

Figure S2. SCLC electron-only devices of $CsPbI_2Br$ layer with varied ETLs.

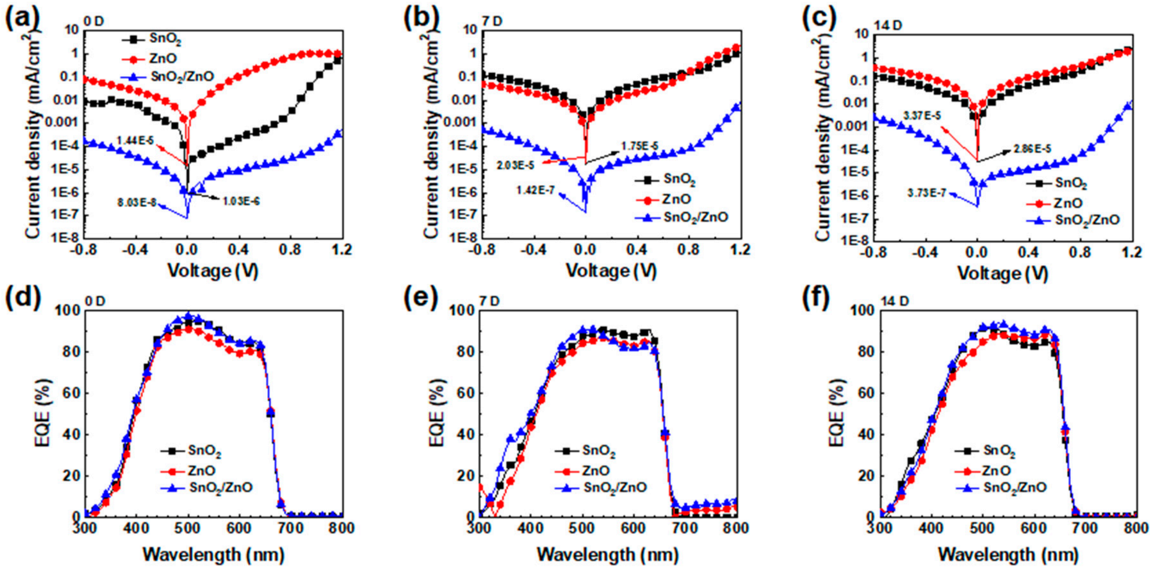

Figure S3. Dark *J-V* curves (a-c) and EQE spectra (d-f) of PD devices for fresh devices (a, d) and aged devices after 7 days (b, e) and 14 days (c ,f) stored in ambient atmosphere.

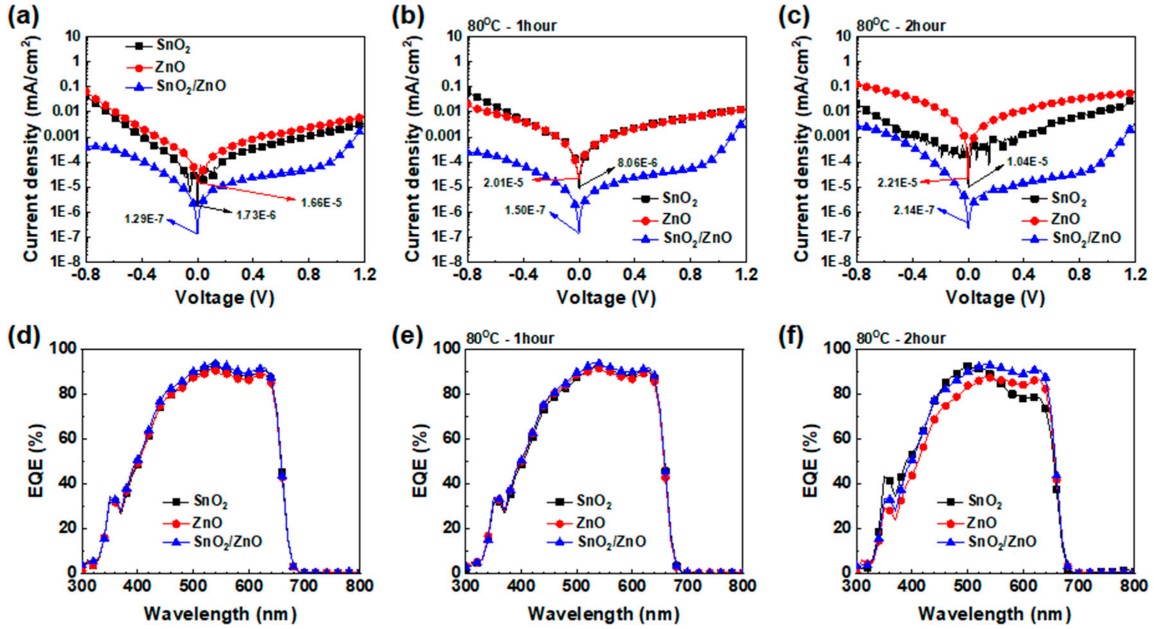

Figure S4. Dark *J-V* curves (a-c) and EQE spectra (d-f) of PD devices with varied ETLs for fresh devices (a, d) and thermally annealed for 1 h (b, e) and 2 h (c, f) at 80 ºC.

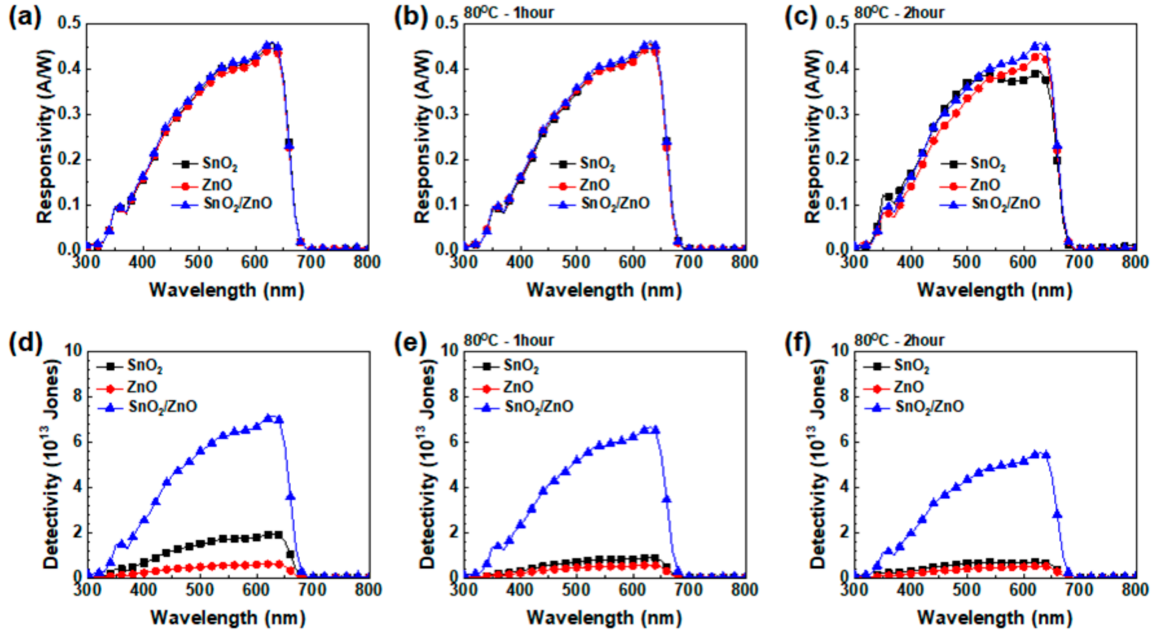

Figure S5. Responsivity (a-c) and specific detectivity (d-f) of PD devices with varied ETLs for fresh devices (a, d) and thermally annealed for 1 h (b, e) and 2 h (c, f) at 80 ºC.
